# Category-Level Object Pose Estimation with Statistic Attention

**DOI:** 10.3390/s24165347

**Published:** 2024-08-19

**Authors:** Changhong Jiang, Xiaoqiao Mu, Bingbing Zhang, Chao Liang, Mujun Xie

**Affiliations:** 1 School of Electrical and Electronic Engineering, Changchun University of Technology, Changchun 130012, China; jch@ccut.edu.cn; 2 School of Mechanical and Electrical Engineering, Changchun University of Technology, Changchun 130012, China; 1202001003@stu.ccut.edu.cn; 3 School of Computer Science and Engineering, Dalian Minzu University, Dalian 116602, China; icyzhang@dlnu.edu.cn; 4 Collage of Computer Science and Engineering, Changchun University of Technology, Changchun 130012, China

**Keywords:** pose estimation, long-range dependencies, higher-order

## Abstract

Six-dimensional object pose estimation is a fundamental problem in the field of computer vision. Recently, category-level object pose estimation methods based on 3D-GC have made significant breakthroughs due to advancements in 3D-GC. However, current methods often fail to capture long-range dependencies, which are crucial for modeling complex and occluded object shapes. Additionally, discerning detailed differences between different objects is essential. Some existing methods utilize self-attention mechanisms or Transformer encoder–decoder structures to address the lack of long-range dependencies, but they only focus on first-order information of features, failing to explore more complex information and neglecting detailed differences between objects. In this paper, we propose SAPENet, which follows the 3D-GC architecture but replaces the 3D-GC in the encoder part with HS-layer to extract features and incorporates statistical attention to compute higher-order statistical information. Additionally, three sub-modules are designed for pose regression, point cloud reconstruction, and bounding box voting. The pose regression module also integrates statistical attention to leverage higher-order statistical information for modeling geometric relationships and aiding regression. Experiments demonstrate that our method achieves outstanding performance, attaining an mAP of 49.5 on the 5°2 cm metric, which is 3.4 higher than the baseline model. Our method achieves state-of-the-art (SOTA) performance on the REAL275 dataset.

## 1. Introduction

Six-dimensional object pose estimation aims to accurately and efficiently estimate the pose and size of objects in a given image, encompassing 3D rotation and 3D translation [1]. This is a fundamental problem in computer vision. Six-dimensional object pose estimation plays a significant role in numerous real-world applications, such as virtual reality [2], scene understanding [3,4], and robotic manipulation [5,6]. Traditionally, pose estimation algorithms rely on handcrafted features to establish correspondences between image templates and images, which struggles with textureless objects and operates at slow speeds. With the rapid development of deep learning methods, deep learning-based pose estimation approaches have emerged. These approaches can be broadly categorized into two main types: instance-level [7,8,9,10,11] and category-level methods [12,13,14,15].

Some instance-level 6D object pose estimation methods achieve high estimation accuracy for given object instances. However, these methods require training on specific instance data, necessitate CAD models of the objects, and can only estimate a limited number of objects with known shapes and textures. In contrast, category-level 6D object pose estimation methods can generalize to unseen objects without the need for CAD models, offering greater flexibility. Category-level methods can be divided into two types: those based on shape priors and those without shape priors. The prior-based estimation methods first extract the shape priors of the objects in an offline mode, then reconstruct their normalized object coordinate space (NOCS) shapes [16], and finally use the Umeyama algorithm [17] to calculate the pose. Although this NOCS shape alignment method can recover the object’s pose, the alignment process is non-differentiable, leading to significant shape errors that affect pose estimation accuracy.

On the other hand, influenced by the development of 3D graph convolution (3D-GC) [18], category-level object pose estimation methods based on 3D-GC have achieved significant breakthroughs. Among these, FS-Net [19] designed a direction-aware 3D-GC autoencoder that learns latent features by reconstructing observations of different objects to estimate their poses. GPV-Pose [14] enhanced the learning of intra-class object shape features by introducing three new branches on the 3D-GC encoder using local geometric relationship constraints and demonstrated outstanding performance. Building on this, HS-Pose [20] proposed a hybrid range latent feature extraction layer that simultaneously perceives both local and global geometric relationships. This layer has improved awareness of translation and scale, making it better suited for handling more complex geometric shapes. However, the inability to capture long-range dependencies limits these methods’ ability to further model geometric relationships. Long-range dependencies are crucial for modeling complex and occluded object shapes. Additionally, perceiving detailed differences between different objects is essential, as these details are vital for distinguishing between objects with similar shapes. Some existing methods use self-attention mechanisms [21,22] or Transformer encoder–decoder structures [23] to compensate for the missing long-range dependencies [24]. However, these methods only focus on the first-order information of the features and fail to extract more complex information. Moreover, they do not pay sufficient attention to the detailed differences between different objects.

**Figure 1:**
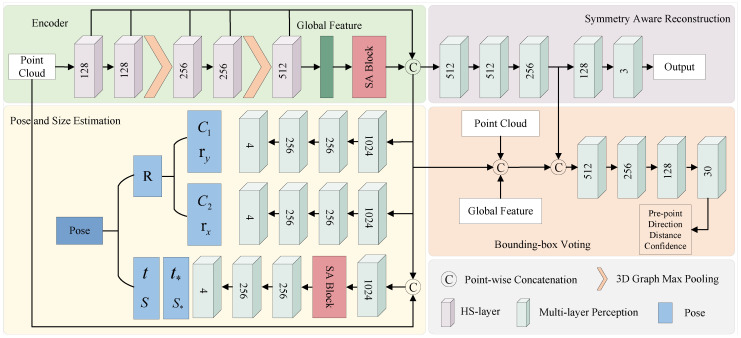
Overview of the proposed SAPENet. It employs the HS-Layer as primary encoder architecture. The core unit of SAPENet is Statistical Attention module, which is integrated at two appropriate stages within the architecture. This module utilizes high-order statistical information to model geometric relationships, capturing long-range dependencies while paying greater attention to the detailed differences between objects. Additionally, three sub-modules are incorporated for pose regression, point cloud reconstruction, and bounding box voting, respectively, forming the complete SAPENet. The pose estimation branch outputs confidence aware rotation $\left\{C_{1},r_{y},C_{2},r_{x}\right\}$, residual translation $t_{*}$, and size $S_{*}$ parameters, from which we recover the pose $\left\{R,t,S\right\}$ in a closed form.

To overcome the limitations of previous methods, we introduce Statistical Attention [25], which utilizes high-order statistical information to model geometric relationships. This approach captures long-range dependencies while emphasizing the detailed differences between objects. To validate the effectiveness of this attention mechanism for pose estimation tasks, we developed a new category-level object pose estimation network called Statistical Attention Pose Estimation Network (SAPENet). Specifically, the structure of this network, illustrated in Figure 1, builds upon the 3D-GC architecture. In the encoder part, the HS-layer replaces the 3D-GC to extract features, and Statistical Attention is added to compute high-order statistical information. The network includes three sub-modules for pose regression, point cloud reconstruction, and bounding box voting. Additionally, the pose regression module incorporates Statistical Attention to strengthen feature modeling by utilizing high-order statistical information. Experiments conducted on the REAL275 dataset demonstrate that SAPENet outperforms existing methods.

The contributions of this paper can be summarized as follows:

- We introduce a statistical attention mechanism capable of capturing long-range feature dependencies and detailed object differences. This attention mechanism addresses critical aspects that were previously lacking in category-level object pose estimation.
- We construct a category-level object pose estimation network, SAPENet, based on 3D-GC, utilizing the introduced statistical attention. By applying statistical attention at various stages, the network models relationships using long-range and high-order information throughout. This network effectively captures the geometric relationships and detailed differences of objects.
- Extensive experiments are conducted on the REAL275 dataset to evaluate SAPENet. The results demonstrate that the proposed method can effectively model complex and detailed object shapes, proving the efficacy of SAPENet in the field of 6D object pose estimation.

## 2. Related Works

### 2.1. Instance-Level 6D Object Pose Estimation

Instance-level 6D object pose estimation tasks require prior knowledge of the object’s CAD model. Based on 3D models, these tasks generally fall into four categories: correspondence-based, template-based, voting-based, and regression-based methods. The correspondence-based method determines the object’s pose by establishing correspondences between input object data and CAD models [26,27]. This approach predicts the overall object pose from local predictions, achieving effective estimation for occluded objects but struggling with texture-less features. Template-based methods, on the other hand, utilize global information rather than local features. They match templates with real images using feature descriptors, akin to an image retrieval task, addressing challenges posed by correspondence-based methods [28,29]. Unfortunately, template-based methods are computationally intensive and memory-consuming, and they fail to match occluded objects. Voting-based methods ascertain confidence through a voting scheme, selecting the pose with the highest confidence [30,31]. However, this process is time-consuming. Lastly, regression-based methods directly estimate the target pose from features, simplifying the inference process and achieving faster speeds [32,33,34]. In summary, instance-level methods necessitate precise CAD models and can only estimate a limited number of object instances, exhibiting limited generalization to unseen objects.

### 2.2. Category-Level 6D Object Pose Estimation

Category-level 6D object pose estimation tasks aim to predict the poses of unseen objects within the same category. Existing category-level methods can be categorized into those based on shape priors and those without shape priors. Shape-prior-based methods, pioneered by NOCS [16], map input features into a unified space and use the Umeyama algorithm to estimate object poses. Subsequent methods have aimed to enhance NOCS shape reconstruction accuracy. For instance, Chen et al. [12] utilized Transformers to model global structural similarities between priors and objects while dynamically adapting to priors using object semantic information. Zou et al. [35] introduced parallel Transformers to learn appearance and geometric features across instances to improve pose estimation. In contrast, shape-prior-free pose estimation methods focus on enhancing pose estimation capabilities from a geometric perspective. FS-Net [19] designed a direction-aware 3D-GC encoder, reconstructing observations of different objects to estimate poses. GPV-Pose [14] introduced decoupled confidence-driven rotation representations, enhancing the learning of category-level pose-sensitive features with geometric insights. Building on this, HS-Pose [20] proposed a hybrid range latent feature extraction layer that perceives both local and global geometric relationships, enhancing translation and scale awareness for handling complex geometric shapes. We further advance geometric relationship modeling by introducing a new attention mechanism to effectively enhance the network’s capability in modeling geometric relationships and details.

## 3. Method

### 3.1. Overview of Network

The proposed SAPENet uses point cloud data as input and consists of four main components: an encoder, a pose regression module, symmetry-based point cloud reconstruction, and bounding box voting. The goal is to estimate the pose and size of category-level objects, enhancing the network’s feature extraction capabilities and its attention to detailed differences.

Specifically, the network structure is illustrated in Figure 1. Firstly, an encoder is utilized as the backbone network to extract features from the input point cloud data. The encoder employs a 3D-GC-based method. To effectively utilize the global geometric relationships within the features, we reference HS-Pose [20] and replace the 3D-GC layers with HS-Layers, which can effectively handle the complex geometric shapes of objects. To further capture long-range dependencies and detailed differences between objects, we introduce statistical attention at the end of the encoder. After obtaining the global features, statistical attention is used to compute the long-range dependencies between features and acquire detailed differences between objects using high-order statistical information. The statistical attention mechanism consists of three steps: computing and normalizing high-order statistics of features, distributing these high-order statistics across the feature map, and adding residual connections. The detailed operations of this module are described in Section 3.2.

Due to the insensitivity of 3D-GC to shape and displacement variations in input point cloud data, the network incorporates three parallel branches: pose regression, symmetry-based point cloud reconstruction, and bounding box voting. In the pose regression module branch, the network predicts both the object’s rotation and translation. Specifically, rotation is predicted in the form of 3D bounding box normal planes. Some normal planes, such as those perpendicular to the object’s surface, are easier to predict. Therefore, two paths are used to estimate the confidence of each normal plane. We let $r_{x},r_{y}$ denote the predicted normal plane and $c_{x},c_{y}$ denote its confidence. To calibrate the plane normals, the network minimizes the following cost function:

(1) $$\begin{array}{cc} & {\hat{\theta }}_{1},{\hat{\theta }}_{2}=argminc_{x}\theta_{1}^{2}+c_{y}\theta_{2}^{2} \end{array}$$

where $\theta_{1}+\theta_{2}+\frac{\pi }{2}=\theta$ represents the angle between the predicted and actual normal planes. The corrected normal planes are then calculated using the Rodrigues rotation formula to obtain the final rotation matrix *R*. For the prediction of translation *t*, the Statistical Attention module is also introduced in this branch to enhance detailed feature information, which is beneficial for accurate translation prediction. During the inference process, only the aforementioned encoder and pose regression module are utilized.

For symmetry-based point cloud reconstruction, we reference GPV-Pose [14], extracting more effective point features using both reflective and rotational symmetries. In the bounding box voting branch, a confidence-weighted point voting strategy is employed to achieve more robust 3D bounding box predictions.

### 3.2. Statistical Attention

**Figure 2:**
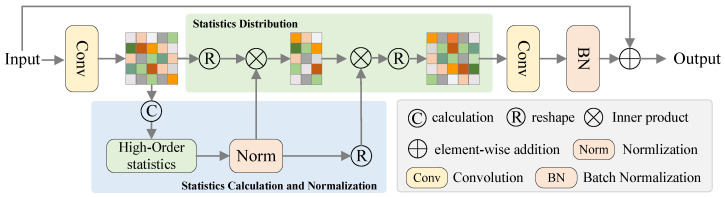
The overall structure of the statistical attention module. The blue shaded area computes statistical data. The green shaded area allocates the statistical data to each position based on weights.

Introducing statistical attention into the backbone and pose regression modules of the SAPENet enables effective modeling of relationships using long-range and high-order information throughout the network. This approach enhances perception of geometric relationships and detailed differences among objects. Statistical attention is implemented as a computationally efficient residual structure, calculating high-order statistics of features and distributing these statistics to corresponding features based on weights to perceive global dependencies across the entire feature set. Due to the inclusion of long-range and higher-order information, the statistical attention mechanism enables explicit modeling of relationships, allowing for the efficient capture of long-range dependencies with reduced computational complexity. Specifically, as illustrated in Figure 2, statistical attention consists of three steps: Computation of high-order statistical metrics of features and normalization. Distribution of these high-order statistics into the feature maps. Addition of residual connections to integrate the enhanced feature representations back into the network.

**Compute high-order statistical moments of the features and normalize them.** Given the input point cloud features $P,P\in R^{N\times D}$, where *N* denotes the number of center points and *D* represents the channel dimension, statistical attention first reduces the dimensionality and decouples inter-channel correlations using a 1D convolution, resulting in new features $X,X\in R^{N\times D^{\prime }}$ with a new channel dimension $D^{\prime }$. These new features are then reinterpreted as a set $Z=\left\{x_{i}|i=1,...,D^{\prime }\right\}$. Subsequently, as illustrated in the blue section of Figure 2, statistical data are computed from the set *Z*. This includes first-order statistics such as mean μ, second-order statistics like variance σ, and higher-order statistics such as skewness $m_{3}$, kurtosis $m_{4}$, etc. For convenience, let us denote this subset of statistical quantities as *B*:

(2) $$\begin{array}{cc} & B=\left\{\mu ,\sigma ,m_{3},m_{4},m_{5},...,\right\} \end{array}$$

Using *B* to model the geometric relationships and detail variances of features, we introduce function M(Z,B) to compute the statistical data of set *Z* within *B*:

(3) $$\begin{array}{cc} & M\left(Z,B\right)=\left\{\varphi_{b}\left(Z\right)|b\in B\right\} \end{array}$$

where $\varphi_{b}\left(Z\right)$ represents the statistical quantities of set *Z*. Subsequently, we perform Euclidean normalization on the statistical quantities in *B* to balance the data scale.

**Allocate statistical quantities.** Using attention mechanisms to allocate statistical data to original features allows focusing on relevant areas with different weights. Depending on semantic information at each position, different statistical data can be assigned. For example, the output $y_{i}$ at position *i* can be formulated as follows:

(4) $$\begin{array}{cc} & Y_{i}=\frac{1}{R(x_{i})}\sum^{b\in M(Z,B)}f\left(x_{i},b\right)\cdot b,i=1,...,D^{\prime } \end{array}$$

where f(·) is the similarity function and $R(x_{i}$) is the normalization factor. Then, we obtain the output $Y=\left[y_{1},...,y_{D^{\prime }}\right]\in R^{C\times D^{\prime }}$.

**Add residual connections.** After assigning statistical data to the original features, the resulting Y is further processed using a 1D convolution to increase its dimensions to match those of the input data. This step facilitates subsequent calculations. Additionally, batch normalization layers are utilized to ensure consistent input–output scaling:

(5) $$\begin{array}{cc} & \hat{Y}=BN\left(Conv\left(Y\right)\right) \end{array}$$

where Conv(·) represents 1D convolution and BN(·) represents the batch normalization layer. Then, a residual connection is added to mitigate the effects of covariance shift:

(6) $$\begin{array}{cc} & \hat{P}=P+\hat{Y} \end{array}$$

where $\hat{P}$ represents the output features.

## 4. Experiments

**Datasets.** We employ the REAL275 [16] dataset for training and testing the proposed network. The REAL275 dataset primarily consists of RGB-D images of 275 objects from real-world scenes, divided into multiple categories, each containing several instances. The objects exhibit diverse poses, appearances, and materials, effectively simulating the complexity found in practical applications. The dataset includes 8000 images, all captured from real-world scenes, ensuring high authenticity and diversity. This diversity is crucial for evaluating the model’s performance in real-world applications.

**Implement Details.** To validate the effectiveness of the proposed network, we follow the methodologies of GPV-Pose [14] and HS-Pose [20] in the experimental setup. In the input stage, based on the Mask R-CNN framework, the segmented results of the targets are transferred to three-dimensional point clouds, which are uniformly sampled with 1028 points selected as the input for the network. Corresponding data augmentation strategies and loss functions are retained. The network is developed using the PyTorch deep learning framework and executed on a computer equipped with 2 NVIDIA GeForce RTX 3090 GPUs. During the model training phase, the Ranger optimizer is employed with a cosine learning rate strategy, setting the initial learning rate to $1e^{-4}$ and weight decay to $5e^{-4}$. The model is trained for a total of 150 iterations with a batch size of 16. Regarding hyperparameter settings, in the statistical attention module, the dimension is reduced from 1024 to 512.

In terms of the choice of orders, an interesting finding emerges: the selection of orders does not affect the weights but only alters the computation method of the weights. No matter how many orders of statistical data are used, it only changes the way the weights are calculated and does not affect the weights themselves. The reason is that the features extracted by the network are fixed, and the statistical attention only calculates the high-order statistics of these features (which can be understood as different ways of expressing the features) without changing them. Therefore, the order of statistical attention does not affect the weight value. Therefore, during training, we use B=[1,2,4,5,6], while during validation, detailed testing is conducted, choosing B=[1,2,3,4,5,6,7] as the final result.

**Evaluation Metrics.** For evaluating the performance metrics, the Average Precision under the union-intersection thresholds of 25, 50, and 75 Intersection over Union (IoU) thresholds is used to estimate object pose and size. Predictions are considered correct if the overlap ratio exceeds the fixed threshold. Metrics for assessing object rotation and translation include 2 cm and 5°. Predictions are deemed correct if errors fall below the specified angle and distance thresholds. Additionally, combinations of rotation and translation thresholds are added, specifically 5°2 cm, 5°5 cm, 10°2 cm, and 10°5 cm.

### 4.1. Ablation Studies

**Impact of different components on performance.** In Table 1, we investigate the effect of various components on model performance. The first row shows the baseline model, which employs the 3D-GC GPV-Pose approach. Replacing the original 3D-GC layer with the HS layer, as seen in the second row, resulted in substantial improvements, with increases of 14.5, 12.3, 13.6, and 9.4 in the 5°2 cm, 5°5 cm, 10°2 cm, and 10°5 cm metrics, respectively. This enhancement is attributed to the HS layer’s ability to exploit global geometric relationships within features, enabling better handling of complex object geometries and thus improving model performance. The final row shows further improvements achieved by integrating the SA module alongside the HS layer. The final model achieved mAP scores of 49.1, 57.5, 72.2, and 84.5 for the 5°2 cm, 5°5 cm, 10°2 cm, and 10°5 cm metrics, respectively, with peak mAP scores of 79.8 for 2 cm and 61.2 for 5°. The introduction of the SA module enhances the model’s ability to capture long-range dependencies and intricate differences between objects by utilizing higher-order statistical information.

**The impact of statistical attention positions.** In this section, the evaluation of statistical attention insertion positions on model performance is presented, as shown in Table 2. The first row in the table shows the result after we replace the original 3D-GC of GPV-Pose with HS-Layer as the baseline model. Then, statistical attention is inserted at five different positions: after the global features in the encoder (Position1), before the global features in the encoder (Position2), before the convolutional block with dimension 1024 in the pose regression module (Position3), after the convolutional block with dimension 1024 in the pose regression module (Position4), and after the first convolutional block with dimension 256 in the pose regression module (Position5), as illustrated in Figure 1. From Table 2, it can be observed that Position1, Position3, and Position4 exhibit the best overall performance. The performance at other positions is slightly lower.

**The impact of dimensions in statistical attention on performance.** This section explores the impact of using 1D convolution to reduce dimensions in statistical attention on model performance. The initial input channel dimension is D=1024. Subsequently, a 1D convolution is used to decrease or increase *D* to decouple channel dimension correlations, resulting in a changed dimension $D^{\prime }$. Table 3 presents the impact of various dimensions on model performance, including 256, 512, 1024, and 2048. It can be observed that the model achieves the optimal overall performance when D=512. The mAP scores for $IoU_{25}$, $IoU_{50}$ and $IoU_{75}$ are 83.0, 82.0, and 75.0, respectively, which are comparable to the baseline model. Specifically, for the 5°2 cm, 5°5 cm, 10°2 cm, and 10°5 cm metrics, the scores are 47.7, 56.5, 70.9, and 84.3, respectively. The model achieves 79.7 and 60.0 in terms of object rotation and translation (2 cm and 5°), respectively. Overall, these results are superior to the baseline model and other configurations, demonstrating the optimal performance.

**The impact of the number of statistical attentions.** The previous ablation analysis on the placement of statistical attention revealed that the models with attention at Position1, Position3, and Position4 achieved better results. In this section, we further compare the impact of the number of statistical attentions using these three optimal positions. The results, as shown in Table 4, analyze the outcomes of adding two modules (Position1+3, Position1+4, and Position3+4) and three modules (Position1+3+4). Overall, the best results were obtained with Position1+4, which involves adding statistical attention before the global features in the encoder and after the convolutional block with a dimension of 1024 in the pose regression module, as illustrated in Figure 1.

**The impact of statistical attention orders.** This section evaluates the impact of using different orders of statistical attention on model performance, as shown in Table 5. Firstly, the second row of the table demonstrates that when only first-order statistical data are used, the model performance already shows significant improvement. This is because the first-order statistic, which includes the global mean, can effectively model each position in the entire model. Then, as the order of statistical data increases, the model performance generally shows an upward trend. Specifically, the model achieves optimal performance, especially in the 10°5 cm metric, with an improvement of 0.5 compared to using only first-order statistics. It is important to note that the change in order was applied only during inference; the same order was used during model training. Finally, further increasing the order does not lead to additional improvements and may even result in a performance decline. This decline could be due to the detrimental effect of excessively high-order statistical information on the model. Therefore, we choose to use all statistical information with an order less than or equal to 7.

**Comparison with different attention modules on the object pose estimation task.** In this section, we compare SAPENet with several classic attention mechanisms, including SE, CBAM, and ECA. For a fair evaluation, we utilize the same backbone network and place the attention modules at identical positions. The results, presented in Table 6, demonstrate that SAPENet outperforms all other attention methods, achieving mAP scores of 83.1, 82.1, and 74.7 in the respective metrics. Specifically, SAPENet attains scores of 49.2, 57.6, 72.7, and 84.7 for the 5°2 cm, 5°5 cm, 10°2 cm, and 10°5 cm metrics, and achieves 80.3 and 61.4 for object rotation and translation (2 cm and 5°), respectively. The superior performance of SAPENet can be ascribed to its integration of higher-order statistical information, unlike previous attention modules. This capability enables SAPENet to model relationships more effectively, capturing long-range dependencies while also focusing on fine-grained differences between objects.

**Comparison of the parameters and computational complexity between the proposed method and HS-Pose.**Table 7 presents a comparison of the parameter count and computational cost between our method and HS-Pose. The first row of the table shows the HS-Pose method, which has 6.1 M parameters and a computational cost of 25.5 G, as calculated by us. Our method adds 0.3 M parameters and 0.7 G to the computational cost compared to HS-Pose. Despite these modest increases in parameters and computational cost, there is a corresponding enhancement in performance. Overall, our method achieves a reasonable balance between cost and performance.

### 4.2. Comparison with State-of-the-Art Methods

Comparison with the state-of-the-art methods on the REAL275 dataset is shown in Table 8. The upper part of the table uses RGB-D-based methods during inference, while the lower part performs pose estimation using depth only. It can be observed that our SAPENet achieves the best performance in four metrics: 5°2 cm, 5°5 cm, 10°2 cm, and 10°5 cm, with scores of 49.2, 57.6, 72.7, and 84.7, respectively. Specifically for the upper part of the table utilizing RGB-D methods, classic approaches such as NOCS, DualPoseNet, SPD, CR-Net, and SGPA are compared. Among these, NOCS achieves the highest mAP of 84.9, likely due to its use of shape priors based on point clouds. Interestingly, our method outperforms NOCS in other metrics without the benefit of shape priors, demonstrating the effectiveness of our approach. Overall, SGPA achieves the best results in the upper part of the table, but there is still some gap compared to our method.

**Figure 3:**
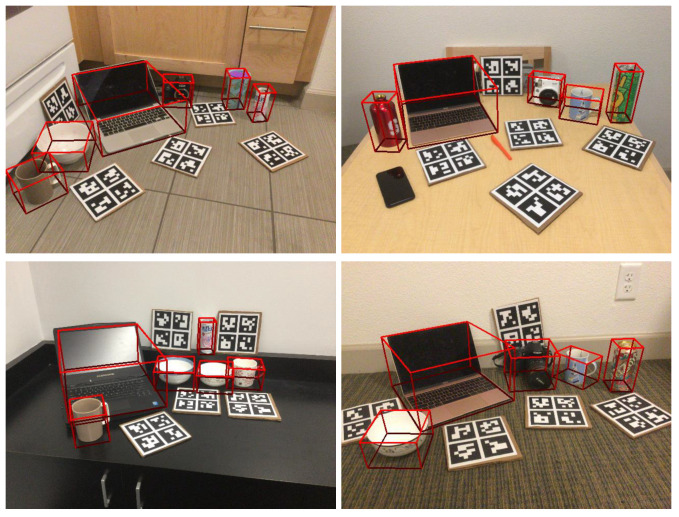
Qualitative results of our method.

Moving to the lower part of the table, which uses depth-only methods for pose estimation, we compare recent approaches such as FS-Net, SAR-Net, RBP-Pose, GPV-Pose, and HS-Pose. GPV-Pose achieves the highest mAP of 83.0, with our method trailing by only 0.9, and our method outperforms GPV-Pose in most metrics. Additionally, HS-Pose achieves the secon dhighest results in metrics 5°2 cm, 5°5 cm, 10°2 cm, and 10°5 cm in the table. However, there is still some difference compared to our method, particularly with SAPENet outperforming HS-Pose by 4.1 in the 10°2 cm metric. These results demonstrate the effectiveness of SAPENet proposed in this paper for pose estimation tasks. We then visualize our approach and the results are shown in Figure 3.

## 5. Conclusions

This paper proposes a category-level 6D object pose estimation method based on 3D-GC named SAPENet. The network applies statistical attention at various stages, leveraging long-range and high-order information to model relationships and enhance the model’s capability to further capture geometric relationships. This approach effectively perceives geometric shapes and subtle differences in objects. The entire network demonstrates superior modeling capabilities for complex and occluded object shapes. Compared to existing methods, our SAPENet achieves state-of-the-art performance. In future work, we aim to further refine the proposed network, explore new attention mechanisms, and enhance accuracy in pose and size predictions.

## Figures and Tables

**Table 1:** Impact of different components on performance.

Configuration	5°2 cm	5°5 cm	10°2 cm	10°5 cm	2 cm	5°
GPV-Pose (baseline)	32.0	42.9	55.0	73.3	69.7	44.7
+HS layer	46.5 (+14.5)	55.2 (+12.3)	68.6 (+13.6)	82.7 (+9.4)	78.2 (+8.5)	58.2 (+13.5)
+ HS layer + SA module	**49.1 (+2.6)**	**57.5 (+2.3)**	**72.2 (+3.6)**	**84.5 (+1.8)**	**79.8 (+1.6)**	**61.2 (+3.0)**

**Table 2:** Comparison of statistical attention positions on the REAL275 dataset.

Configuration	${\mathrm{IoU}}_{25}$	${\mathrm{IoU}}_{50}$	${\mathrm{IoU}}_{75}$	5°2 cm	5°5 cm	10°2 cm	10°5 cm	2 cm	5°
baseline	**83.1**	**82.1**	75.0	46.1	55.2	69.9	84.3	78	58.5
Position1	**83.1**	81.2	**75.1**	**47.3**	55.4	**71.8**	84.3	79.7	58.6
Position2	**83.1**	81.5	74.9	45.7	55.2	70.8	84.7	78.5	58.2
Position3	83.0	81.2	74.8	47.0	56.4	71.4	**84.6**	79.5	59.3
Position4	83.0	82.0	75.0	47.7	**56.5**	70.9	84.3	79.7	**60.0**
Position5	**83.1**	81.1	**75.1**	46.1	54.3	71.4	84.1	**79.8**	57.6

**Table 3:** Comparison of statistical attention dimensions on the REAL275 dataset.

Configuration	${\mathrm{IoU}}_{25}$	${\mathrm{IoU}}_{50}$	${\mathrm{IoU}}_{75}$	5°2 cm	5°5 cm	10°2 cm	10°5 cm	2 cm	5°
baseline	**83.1**	**82.1**	**75.0**	46.1	55.2	69.9	84.3	78.0	58.5
256	**83.1**	81.3	74.3	46.1	55.3	69.6	83.5	78.3	58.6
512	83.0	82.0	**75.0**	**47.7**	**56.5**	70.9	84.3	**79.7**	**60.0**
1024	**83.1**	81.3	74.7	46.6	55.2	70.9	84.7	78.1	58.5
2048	83.0	81.8	74.8	46.8	55.7	**71.9**	**85.1**	79.5	59.0

**Table 4:** Comparison of the number of statistical attentions on the REAL275 dataset.

Configuration	${\mathrm{IoU}}_{25}$	${\mathrm{IoU}}_{50}$	${\mathrm{IoU}}_{75}$	5°2 cm	5°5 cm	10°2 cm	10°5 cm	2 cm	5°
baseline	**83.1**	**82.1**	**75.0**	46.1	55.2	69.9	84.3	78.0	58.5
Position1+3	**83.1**	81.7	74.9	46.8	56.7	71.0	**85.8**	78.0	60.1
Position1+4	**83.1**	82.0	74.7	**49.1**	**57.5**	**72.2**	84.5	**79.8**	**61.2**
Position3+4	83.0	81.6	74.4	47.1	55.6	71.6	84.9	79.0	58.7
Position1+3+4	**83.1**	81.0	70.1	41.1	51.7	66.3	82.3	74.6	55.1

**Table 5:** Comparison of statistical attention orders on the REAL275 dataset.

μ	σ	$m_{3}$	$m_{4}$	$m_{5}$	$m_{6}$	$m_{7}$	$m_{8}$	${\mathrm{IoU}}_{25}$	${\mathrm{IoU}}_{50}$	${\mathrm{IoU}}_{75}$	5°2 cm	5°5 cm	10°2 cm	10°5 cm	2 cm	5°
								83.1	**82.1**	75.0	46.1	55.2	69.9	84.3	78.0	58.5
√								83.1	**82.1**	74.6	49.4	57.3	72.5	84.2	80.4	61.0
√	√							83.1	81.9	74.7	49.1	57.1	72.1	84.1	80.0	61.0
√	√	√						83.1	81.9	74.6	49.3	57.4	72.3	84.2	80.2	61.2
√	√		√					83.1	81.9	**74.8**	**49.5**	57.5	72.2	84.2	80.1	61.2
√	√	√	√					83.1	82.0	74.7	49.1	57.4	71.9	84.3	79.7	61.1
√	√		√	√				83.1	81.9	74.5	48.8	56.8	72.2	84.1	79.9	60.7
√	√	√	√	√				83.1	82.0	74.3	49.1	57.4	71.8	84.1	79.7	61.2
√	√		√	√	√			83.1	82.0	**74.8**	49.3	57.5	72.3	84.2	80.1	61.5
√	√	√	√	√	√			83.1	81.9	74.6	49.2	57.0	72.5	84.1	**80.7**	60.7
√	√		√	√	√	√		83.1	81.9	**74.8**	**49.5**	57.4	72.5	84.1	80.3	61.1
√	√	√	√	√	√	√		83.1	**82.1**	74.7	49.2	**57.6**	**72.7**	**84.7**	80.3	**61.4**
√	√	√	√	√	√	√	√	83.1	81.9	74.7	49.4	57.4	72.4	84.4	80.2	61.2

**Table 6:** Comparison with different attention modules on the object pose estimation task.

Attention	${\mathrm{IoU}}_{25}$	${\mathrm{IoU}}_{50}$	${\mathrm{IoU}}_{75}$	5°2 cm	5°5 cm	10°2 cm	10°5 cm	2 cm	5°
+SE	83.1	81.7	74.9	46.6	55.5	70.1	83.4	79	59.1
+CBAM	82.8	81.5	74.7	47.2	55.4	70.8	84.0	79.4	59.3
+ECA	83.0	81.2	74.8	47.1	56.4	70.8	84.2	79.5	59.3
SAPENet (ours)	**83.1**	**82.1**	**74.7**	**49.2**	**57.6**	**72.7**	**84.7**	**80.3**	**61.4**

**Table 7:** Comparison of the parameters and computational complexity between the proposed method and HS-Pose.

Method	2 cm	5°	Para.	Gflops
HS-Pose	78.2	58.2	6.1 M	25.5
SAPENet	**79.8**	**61.2**	**6.4 M**	**26.2**

**Table 8:** Comparison with the state-of-the-art methods on the REAL275 dataset. The best results are highlighted in bold.

Method	${\mathrm{IoU}}_{25}$	${\mathrm{IoU}}_{50}$	${\mathrm{IoU}}_{75}$	5°2 cm	5°5 cm	10°2 cm	10°5 cm
NOCS [16]	**84.9**	80.5	30.1	7.2	10.0	13.8	25.2
DualPoseNet [36]	-	79.8	62.2	29.3	35.9	50.0	66.8
SPD [37]	83.4	77.3	53.2	19.3	21.4	43.2	54.1
CR-Net [38]	-	79.3	55.9	27.8	34.3	-	60.8
SGPA [12]	-	80.1	61.9	35.9	39.6	61.3	70.7
FS-Net [19]	84.0	81.1	63.5	19.9	33.9	-	69.1
SAR-Net [39]	-	79.3	62.4	31.6	42.3	50.3	68.3
RBP-Pose [40]	-	-	67.8	38.2	48.1	63.1	79.2
GPV-Pose [14]	84.1	**83.0**	64.4	32.0	42.9	55.0	73.3
HS-Pose [20]	84.2	82.1	**74.7**	46.5	55.2	68.6	82.7
SAPENet(ours)	83.1	82.1	**74.7**	**49.2**	**57.6**	**72.7**	**84.7**

## Data Availability

The original data presented in the study are openly available in https://geometry.stanford.edu/projects/NOCS_CVPR2019/, accessed on 13 August 2024.
